# Comparison of early changes in and factors affecting vault following posterior chamber phakic Implantable Collamer Lens implantation without and with a central hole (ICL V4 and ICL V4c)

**DOI:** 10.1186/s12886-016-0336-8

**Published:** 2016-09-07

**Authors:** Xun Chen, Huamao Miao, Rajeev Krishnan Naidu, Xiaoying Wang, Xingtao Zhou

**Affiliations:** 1 Key Lab of Myopia, Ministry of Health, Shanghai, China; 2 EYE & ENT Hospital of Fudan University, Shanghai, China; 3 School of Medicine, The University of Sydney, Camperdown, NSW Australia; 4 Department of Ophthalmology, Myopia Key Laboratory of the Health Ministry, Eye and ENT Hospital of Fudan University, No. 19 BaoQing Road, Shanghai, 200031 China

**Keywords:** Implantable Collamer Lens, Vault, Pupil

## Abstract

**Background:**

To objectively compare the early changes in vault over time following implantation of an Implantable Collamer Lens without (ICL V4) and with (ICL V4c) a central hole and the respective factors affecting vault change in moderate to high myopia.

**Methods:**

This prospective study comprised of 38 eyes of 38 patients implanted with ICL V4 and 39 eyes of 39 patients implanted with ICL V4c intraocular lenses. We quantitatively assessed the postoperative values of vault and pupil size at 1 day, 1 week, and 1 month following implantation using a rotating Scheimpflug camera (Pentacam). We compared these postoperative values within and between the two groups and identified the factors affecting vault change.

**Results:**

The mean vaults at 1 day, 1 week, and 1 month following ICL V4 implantation were 303.68 ± 185.11, 517.89 ± 160.07 and 521.32 ± 155.72 μm respectively, and those following ICL V4c were 316.67 ± 186.89, 495.13 ± 180.84 and 510.77 ± 175.51 μm, respectively. There was a significant difference in vault between 1 day and 1 week postoperatively. There was a significant association between the vault change and the pupil size change in both groups from 1 day to 1 month postoperatively (Pearman correlation coefficient; ICL V4: *r* = 0.585, *P* = 0.001; ICL V4c: *r* = 0.588, *P* <0.001). The vault value 1 month after implantation of ICL V4 and ICL V4c was associated with the preoperative anterior chamber depth, horizontal corneal diameter, horizontal and vertical sulcus-to-sulcus.

**Conclusions:**

Pupil movement is a critical factor in vault change, with increasing vault observed postoperatively from 1 day to 1 week associated with the declining effects of pharmacological miosis and increasing pupil size. The anterior chamber depth, horizontal corneal diameter, horizontal and vertical sulcus-to-sulcus show some correlation with vault.

**Electronic supplementary material:**

The online version of this article (doi:10.1186/s12886-016-0336-8) contains supplementary material, which is available to authorized users.

## Background

The Visian Implantable Collamer Lens (ICL, Staar Surgical Co.) is a posterior chamber phakic intraocular lens (pIOL) that has been reported to be a safe and effective surgical option for the correction of moderate to high myopia [1, 2]. Although the ICL offers outstanding advantages, there have been reports in the literature of postoperative complications associated with both high and low degrees of vault of the ICL over the crystalline lens. Low vault may lead to mechanical contact with the crystalline lens or inadequate aqueous circulation, which is responsible for a high incidence of anterior capsular opacification and cataract formation [2–4]. Conversely, excessively high vault causes mechanical contact between the ICL and the iris, resulting in inflammation, high intraocular pressure, angle-closure glaucoma and pigment dispersion syndrome [5].

Recently, a new implantable collamer lens (ICL V4c) has become available, with a 360 μm central hole that allows for the natural flow of aqueous humor without the need for a peripheral iridotomy. This lens provides excellent visual performance almost equivalent to that of the conventional phakic IOL (ICL V4) and may reduce the risk of anterior capsular opacification and cataract formation [6–11].

Previous studies [12–15] have shown that the pupil constriction in response to light can affect the vault, eventually causing the ICL to move posteriorly towards the crystalline lens, leading to a significant decrease in central vault under photopic conditions. Du et al. [16] reported that the distance between the ICL and the crystalline lens reduced as the ICL was moved posteriorly by the iris as a result of pupil constriction during pharmacologic accommodation with topical pilocarpine. Simultaneously, the anterior surface of the crystalline lens became more convex and moved anteriorly, further reducing the central vault of the ICL.

To the best of our knowledge, there is currently limited literature available regarding early changes in and factors affecting vault following conventional and hole ICL implantation surgery. Considering that intraoperative miotic agents have a relatively long-term effect on pupil constriction, there is potentially significant effects on early vault change following implantation of ICL V4 and ICL V4c pIOLs. The purpose of the present study was to evaluate the early changes in vault in the first month following ICL V4 and ICL V4c implantation using a rotating Scheimpflug camera (Pentacam) and assess the correlation between vault changes and pupil size changes and investigate any other relevant factors affecting vault.

## Methods

### Study population

This study was conducted in accordance with the principles of the Declaration of Helsinki and was approved by the Ethics Committee of the Eye and ENT Hospital Review Board of Fudan University. Written informed consent was obtained from all patients after the nature and possible consequences of the study were explained.

Thirty-eight eyes of 38 patients (12 males and 26 females) implanted with the ICL V4 pIOLs and 39 eyes of 39 patients (12 males and 27 females) implanted with the ICL V4c lens were included in this study. All patients were treated for the correction of moderate to high myopia at the Eye and ENT Hospital of Fudan University, Shanghai, People’s Republic of China.

### Preoperative evaluation

A comprehensive ophthalmic examination was performed preoperatively on all patients. The examinations included uncorrected distance visual acuity (UDVA) and corrected distance visual acuity (CDVA) using a Snellen chart, manifest spherical and cycloplegic refractions, slit-lamp biomicroscopic and fundoscopic examinations, intraocular pressure (IOP, non-contact tonometer), corneal topography (Pentacam, Oculus, Germany), central corneal thickness (Pentacam), horizontal corneal diameter (white-to-white, WTW, IOL master, Carl Zeiss, Germany), axial length (A-scan ultrasound), anterior chamber depth (ACD, Pentacam, measured from the corneal endothelium to the anterior lens), corneal endothelial cell density (ECD, noncontact specular microscopy, SP-2000P, Topcon Corporation, Japan), optical coherence tomography (OCT, Optovue, USA), and ultrasound biomicroscopy (UBM, Quantel medical, France).

Inclusion criteria for this study included patient age between 18 and 45 years, myopia between −0.50 and −21.00 DS, astigmatism between 0 and −6.00 DC, anterior chamber depth (ACD) of 2.80 mm or more, and an endothelial cell density greater than 2000 cells/mm^2^. Patients were also required to have a reasonable expectation of surgical outcomes, no preexisting ocular pathology, no previous keratoconus, cataract or glaucoma, and no systemic disease.

### Implantable Collamer Lens calculation

The ICL is a plate-haptic single-piece intraocular lens made of collamer, which is a flexible, hydrophilic material consisting of HEMA Hydrogel, water, and porcine collagen. It can be folded and implanted in the posterior chamber via a 2.8–3.2 mm corneal incision. It has a high degree of biocompatibility, good permeability of gases and metabolites, and good absorption of ultraviolet radiation. The ICL design has been modified many times in the past. In this study, the phakic IOL patients were implanted with either the ICL V4 or the ICL V4c lens designs. The ICL V4 is a 6.0 mm wide lens and comes in four sizes (11.5, 12.0, 12.5 and 13.0 mm in length). The lens has a central convex–concave optic zone with a diameter of 4.5–6.0 mm, a spherical power range of −3.00 to −23.00 DS and a cylindrical power range of +1.00 to +6.00 DC. The ICL V4c is a 6.00 mm wide lens and comes in four sizes (12.1, 12.6, 13.2 and 13.7 mm in length). Its optic zone diameter is 4.9–5.8 mm, with a spherical power range of - 0.50 to - 18.00 DS and a cylindrical power range of +0.50 to +6.00 DC.

ICL power calculations were performed by the manufacturer (STAAR Surgical) using a modified vertex formula. The variables in the formula included preoperative manifest spherical and cycloplegic refractions, keratometric power, central corneal thickness and central ACD. The size (length) of the implanted ICL was determined based on the patient’s WTW and ACD. In principle for the ICL V4, when the ACD was 2.80 ~ 3.00 mm, ICL length = WTW + 0.2 ~ 0.4 mm; when the ACD was 3.00 ~ 3.50 mm, ICL length = WTW + 0.4 ~ 0.6 mm; when the ACD was 3.50 ~ 3.70 mm, ICL length = WTW + 0.6 ~ 0.8 mm. For the ICL V4c, the sizes (lengths) of 12.1, 12.6, 13.2 and 13.7 mm were equal to the ICL V4 sizes (lengths) of 11.5, 12.0, 12.5 and 13.0 mm, respectively.

### ICL surgical procedure

Preoperative peripheral iridotomies by neodymium - yttrium - aluminum - garnet (Nd:YAG) laser or intraoperative peripheral iridotomies were performed for ICL V4 implantations and no peripheral iridotomies were performed for ICL V4c implantations. On the day of surgery, all patients were administered with dilating and cycloplegic agents (2.5 % phenylephrine and 1 % tropicamide, Alcon, China). After topical anaesthesia (0.4 % oxybuprocaine hydrochloride, Santen, Japan) and injection of a viscoelastic surgical agent (1.7 % Sodium hyaluronate; Bausch & Lomb, China) into the anterior chamber, an ICL V4 IOL was inserted via a 2.8–3.2 mm temporal clear corneal incision with the use of an injector cartridge (STAAR Surgical). After the ICL was placed in the posterior chamber, the surgeon then completely removed the viscoelastic surgical agent from the eye using a balanced salt solution and instilled a miotic agent (0.005 % carbachol, Bausch & Lomb, China). All surgeries were uneventful and no intraoperative complications were observed. Following surgery, a combination antibacterial and steroidal medication (0.1 % Tobramycin dexamethasone, Alcon, China) was prescribed four times daily for 3 days followed by fluorometholone eyedrops tapered gradually over 2 weeks. Antibiotic eyedrops (0.5 % left Ofloxacin, Santen, Japan) were then prescribed four times daily for 1 week, along with non-steroidal anti-inflammatory eyedrops (NSAID, pranoprofen, Senju, Japan) four times daily for 2 weeks, and artificial tears four times daily for 1 month.

### Measurement of vault

The central vault is the distance between the back surface of the ICL and the front surface of the crystalline lens. It was quantitatively measured postoperatively at 1 day, 1 week, and 1 month after surgery using a rotating Scheimpflug camera (Pentacam, Oculus, Germany) with the patient’s chin placed on the chin rest and the forehead against the forehead strap. The patient was asked to open both eyes and fixate on the blue fixation target in the center of the black background. Twenty-five Scheimpflug images were automatically recorded within 2 s once perfect alignment was obtained. Image quality was checked using the quality factor value for each eye. An experienced examiner who was blind to the patients measured the central vault value from the Pentacam overview diagram using the image analysis software program accompanying the device. The vault was measured centered on the optical axis, which was shown as a white dotted line on the screen. The pupil size was automatically measured by the Pentacam device. All measurements of the ICL V4 and ICL V4c vault were taken under the same mesopic conditions.

### Statistical analysis

All statistical analyses were performed using SPSS version 20.0 (SPSS Inc., IBM, USA) and results are expressed as mean ± standard deviation (SD). The Kolmogorov-Smirnov test was chosen to confirm the normal distribution of the data. For comparison between eyes with ICL V4c versus ICL V4 implantation, independent t tests were conducted for parameters with continuous variables. Repeated measures analysis of variance (ANOVA) with bonferroni-adjusted post hoc comparisons was performed to evaluate differences in variables post operation. Stepwise multiple regression analysis was performed to investigate the correlation between several variables and the vault at 1 month after surgery. The dependent variable was the central vault, and the independent variables included patient age, preoperative refraction, horizontal corneal diameter (WTW), horizontal and vertical sulcus-to-sulcus (STS) distance, ACD, axial length, keratometric readings and ICL power. *P* <0.05 was considered statistically significant.

## Results

The demographics of the preoperative parameters are summarized in Table 1. All surgical procedures were uneventful and no postoperative complications were observed during the observation period. The postoperative changes in vault and pupil size of all eyes after implantation with ICL V4 and ICL V4c pIOLs are presented in Table 2.

**Table 1:** Demographics of preoperative parameters

Parameter	ICL V4	ICL V4c	*P* value
*N*, eyes	38	39	
Age, years	28.66 ± 7.53(20 ~ 45)	28.69 ± 6.44(20 ~ 45)	0.98
UDVA (Snellen lines)	0.06 ± 0.04(0.01 ~ 0.15)	0.07 ± 0.06(0.01 ~ 0.15)	0.29
CDVA (Snellen lines)	0.94 ± 0.17(0.50 ~ 1.20)	0.97 ± 0.15(0.7 ~ 1.20)	0.39
Refractive errors (D)			
Spherical	−11.97 ± 3.46(−5.25 ~ −20.50)	−11.72 ± 3.58(−7.25 ~ −20.25)	0.76
Cylindrical	−1.34 ± 1.02(0 ~ −4.75)	−1.47 ± 1.10(−0.25 ~ −5.00)	0.59
Spherical equivalent	−12.64 ± 3.60(−5.75 ~ −21.38)	−12.46 ± 3.67(−7.75 ~ −22.00)	0.83
Keratometric value (D)			
Flat K	43.39 ± 1.73(38.3 ~ 46.7)	43.12 ± 1.74(39.4 ~ 46.7)	0.51
Steep K	44.60 ± 2.03(38.7 ~ 49.2)	44.52 ± 1.87(41.2 ~ 49.0)	0.86
STS (mm)			
Vertical	12.36 ± 0.43(11.54 ~ 13.33)	12.36 ± 0.68(10.67 ~ 13.75)	0.98
Horizontal	11.76 ± 0.37(11.12 ~ 12.64)	11.81 ± 0.62(10.31 ~ 13.39)	0.68
IOP (mm Hg)	14.08 ± 2.38(8.8 ~ 19.1)	15.20 ± 2.70(9.9 ~ 20.1)	0.57
Axial length (mm)	28.24 ± 1.78(25.01 ~ 31.49)	28.24 ± 1.78(25.42 ~ 32.19)	0.99
WTW diameter (mm)	11.77 ± 0.34(11.2 ~ 12.5)	11.85 ± 0.49(10.7 ~ 12.7)	0.39
ACD (mm)	3.21 ± 0.23(2.83 ~ 3.70)	3.18 ± 0.19(2.87 ~ 3.70)	0.86
CCT (mm)	521.08 ± 35.70(467 ~ 603)	515.51 ± 22.26(469 ~ 572)	0.12
ECD (cells/mm^2^)	3254.18 ± 366.74(2331 ~ 3951)	3270.18 ± 351.21(2374 ~ 4258)	0.85
Pupil size (mm)	3.38 ± 0.74(2.09 ~ 4.48)	3.14 ± 0.60(2.14 ~ 4.42)	0.52
ICL size (mm)	12.36 ± 3.84(11.5 ~ 13.0)	12.97 ± 0.47(12.1 ~ 13.7)	
ICL power (D)	−15.16 ± 7.22(−8.50 ~ −23.00)	−12.62 ± 2.92(−8.50 ~ −18.00)	

Results are expressed as means ± standard deviation (range)

*N* number of eyes, *UDVA* uncorrected distance visual acuity, *CDVA* corrected distance visual acuity, *D* diopters, *K* keratometry, *STS* sulcus to sulcus, *IOP* intraocular pressure, *WTW* horizontal white-to-white diameter, *ACD* anterior chamber depth, *CCT* central corneal thickness, *ECD* corneal endothelial cell density, *ICL* implantable collamer lens

**Table 2:** Postoperative vault and pupil size in eyes undergoing implantation of ICL V4 and ICL V4c

Parameter	ICL V4			ICL V4c		
Time course	1 day	1 week	1 month	1 day	1 week	1 month
Pupil size (mm)						
Mean ± SD	1.82 ± 0.66	3.50 ± 0.70	3.59 ± 0.68	2.43 ± 0.76	3.26 ± 0.45	3.31 ± 0.53
Range	1.02 ~ 3.63	2.01 ~ 5.54	2.31 ~ 5.52	1.21 ~ 3.95	2.11 ~ 4.75	2.23 ~ 4.84
Vault (um)						
Mean ± SD	303.68 ± 185.11	517.89 ± 160.07	521.32 ± 155.72	316.67 ± 186.89	495.13 ± 180.84	510.77 ± 175.51
Range	0 ~ 680	270 ~ 920	290 ~ 980	0 ~ 690	150 ~ 850	190 ~ 860

*ICL* implantable collamer lens, *SD* standard deviation

**Fig. 1:**
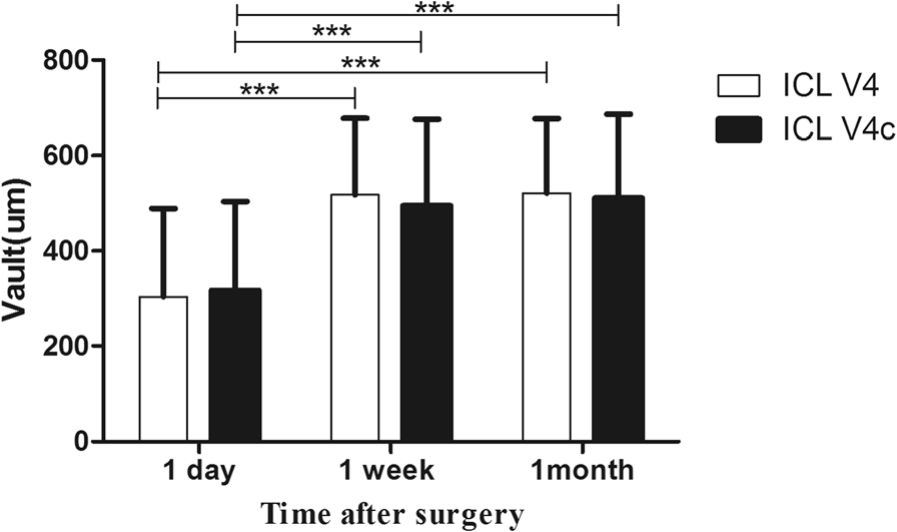
Time course of the phakic IOL vaults in the two groups following implantation of ICL V4 and ICL V4c. (***P <0.001)

The time courses of the phakic IOL vaults in the two groups are shown in Fig. 1. For the two groups, there were significant differences between the vault at 1 day and 1 week after surgery (*P* <0.001) and the vault between 1 day and 1 month (*P* <0.001). There was no significant difference between the vault at 1 week and 1 month after surgery (*P* = 1.00, *P* = 0.07, respectively). There was also no significant difference in vault between the ICL V4 and ICL V4c groups postoperatively.

**Fig. 2:**
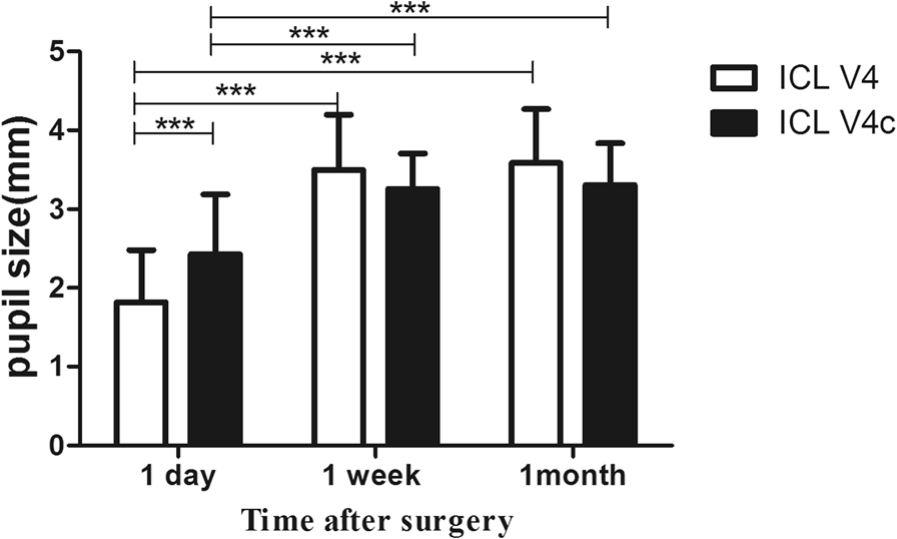
Time course of pupil size in the two groups following implantation of ICL V4 and ICL V4c. (***P <0.001)

The time course of pupil size change in the two groups is shown in Fig. 2. For the two groups, there were significant differences between the values at 1 day and 1 week after surgery (*P* <0.001), and between 1 day and 1 month after surgery (*P* <0.001). There was no significant difference in pupil size between 1 week and 1 month after surgery (*P* = 1.00). There was also a significant difference in pupil size between the ICL V4 and ICL V4c groups postoperatively at 1 day (*P* <0.001), however there was no significant difference between the two groups postoperatively at 1 week or 1 month (*P* = 0.10).

**Fig. 3:**
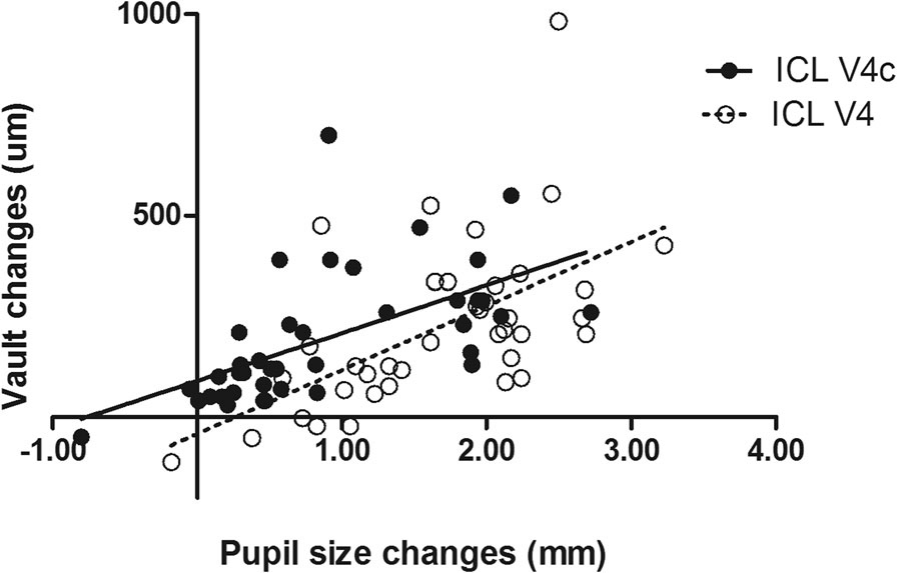
Correlation of vault changes and pupil size changes in the ICL V4 and ICL V4c groups from 1 day postoperatively to 1 month postoperatively. (Pearman correlation coefficient; ICL V4: *r* = 0.585, *P* = 0.001, y = 159.0 x − 51.3; ICL V4c: *r* = 0.588, *P* <0.001, y = 118.5 x + 89.8, respectively)

The correlation between the short-term changes in vault and pupil size immediately after surgery were evaluated. There was a significant association between the vault changes and the pupil size changes in both the ICL V4 and ICL V4c groups from 1 day postoperatively to 1 month postoperatively. (Pearman correlation coefficient; ICL V4: *r* = 0.585, *P* =0.001; ICL V4c: *r* = 0.588, *P* <0.001, respectively) (Fig. 3).

**Fig. 4:**
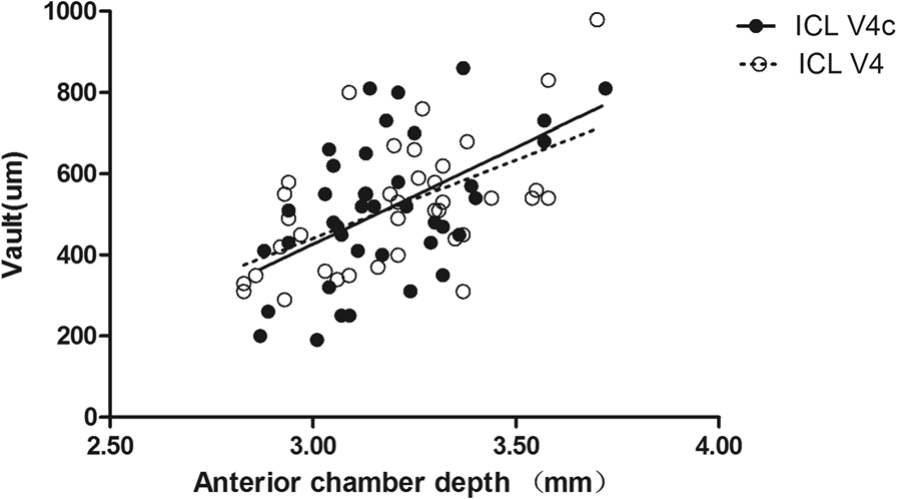
Correlation between anterior chamber depth and the central vault in the ICL V4 and ICL V4c groups (*r* = 0.562, *P* <0.001; *r* = 0.524, *P* = 0.001, respectively)

**Fig. 5:**
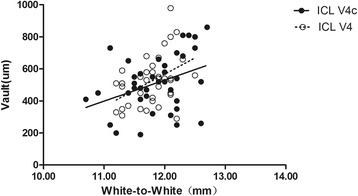
Correlation between white-to-white and the central vault in the ICL V4 and ICL V4c groups (*r* = 0.440, *P* = 0.006; *r* = 0.366, *P* = 0.022, respectively)

**Fig. 6:**
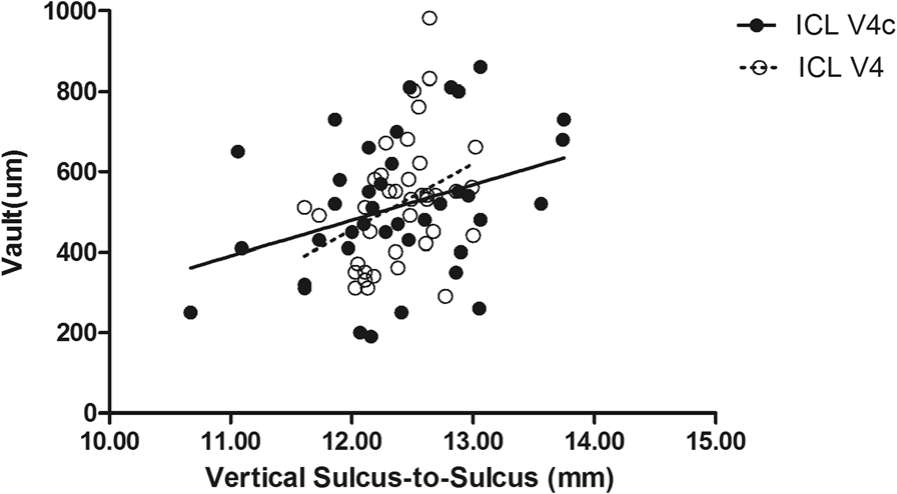
Correlation between horizontal sulcus to sulcus and the central vault in the ICL V4 and ICL V4c groups (*r* = 0.355, *P* = 0.029; *r* =0.365, *P* = 0.022, respectively)

**Fig. 7:**
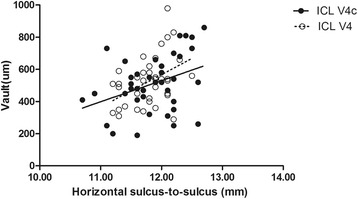
Correlation between vertical sulcus to sulcus and the central vault in the ICL V4 and ICL V4c groups (*r* = 0.345, *P* = 0.034; *r* = 0.344, *P* = 0.032, respectively)

The results of multiple regression analyses are shown in Table 3. The independent variable relevant to the vault in the two group was ACD (*R*^2^ = 0.357, *P* <0.001; *R*^2^ = 0.316, *P* = 0.001, respectively). The multiple regression equation was expressed as follows: ICL V4 Central vault (μm) = (386.51 * ACD) - 718.77, ICL V4c Central vault (μm) = (503.43 * ACD) - 1075.64, respectively. The standardized partial regression coefficient was calculated to determine the magnitude of each variable’s influence. Apart from ACD, the WTW, horizontal and vertical STS were also relevant variables to the vault in the two groups. The central vault was positively correlated with WTW of the two groups (Pearman correlation coefficient; ICL V4: *r* = 0.440, *P* = 0.006; ICL V4c: *r* = 0.366, *P* = 0.022, respectively), horizontal STS (ICL V4: *r* = 0.355, *P* = 0.029; ICL V4c: *r* =0.365, *P* = 0.022, respectively), and vertical STS (ICL V4: *r* = 0.345, *P* = 0.034; ICL V4: *r* = 0.344, *P* = 0.032, respectively). The relationships between vault and ACD, WTW, horizontal and vertical STS are shown in Figs. 4, 5, 6 and 7, respectively. There was no significant correlation shown with any other factors.

**Table 3:** Results of Stepwise Multiple Regression Analyses of ICL V4 and ICL V4c

Variables	Partial Regression Coefficient	Standardized Partial Regression Coefficient	*P* Value
ICL V4			
Anterior chamber depth (mm)	386.51	0.562	<0.001
Constant	−718.77		0.02
*R* ^2^ = 0.357, Adjusted *R* ^2^ = 0.320			
ICL V4c			
Anterior chamber depth (mm)	503.43	0.518	0.001
Constant	−1075.64		0.02
*R* ^2^ = 0.316, Adjusted *R* ^2^ = 0.297

## Discussion

Implantable collamer lenses are a safe and effective phakic IOL option for the surgical correction of moderate to high myopia. In the present study, we aimed to assess the early changes in phakic IOL vault in the first month immediately after implantation of ICLs, both without and with a central hole. We measured patient age, preoperative refraction, horizontal corneal diameter (WTW), horizontal and vertical sulcus to sulcus distance, ACD, axial length, keratometric readings, ICL power, postoperative phakic IOL vault and pupil size, the phakic IOL vault changes and the pupil size changes of eyes following implantation of ICL V4 and ICL V4c IOLs. Our results demonstrated a significant increase in vault postoperatively from 1 day to 1 week after implantation of both ICL V4 and ICL V4c IOLs, which then remained stable from 1 week onwards in both groups. We found that the change in pupil size was a critical factor to the vault change, and postoperative vault was associated with preoperative ACD, WTW, horizontal and vertical STS in eyes implanted with ICL V4 and ICL V4c. In recent studies, rotating Scheimpflug imaging (Pentacam) [17–19], anterior segment OCT [4, 13, 20, 21], and UBM [22] have been used to measure the phakic IOL vault and have all provided accurate and reproducible measures of anterior segment biometry. In our present study, we used the rotating Scheimpflug imaging system (Pentacam) that has proved to be a repeatable measurement to evaluate the vault [17].

Previous studies [4, 5, 17–19, 23] investigating vault following ICL implantation have demonstrated a tendency for the vault to decrease slightly in the long term for both conventional ICLs and hole ICLs. Currently, there is limited literature available investigating changes in vault as early as 1 day following implantation of ICL V4c IOLs. One study by Kamiya et al. [17] reported the comparison of vault following implantation of both ICL V4 and ICL V4c from 1 week to 6 months postoperatively. This study, however, measured the central vault when the pupil was dilated and vault 1 day after the surgery was not measured, so the vault likely have increased slightly due to a larger pupil size. In contrast, the design and outcomes of our present study more closely reflect the natural process after surgery. Another study by Kojima et al. [21] investigating vault changes after the implantation of conventional ICLs over a 1-year period demonstrated a statistically significant decrease in vault from 1 day to 3 months after surgery, with vault stabilizing after 3 months. This study, however, did not report the effects of changes in pupil size on vault, in particular at 1 day postoperatively, which may partly explain the difference in results between their findings and ours. This is important as the prolonged effects of the miotic agents used intraoperatively are likely to have contributed to the short term changes in vault due to pupil size changes, with our study demonstrating a statistically significant difference in postoperative pupil size between 1 day and all other time points after both ICL V4 and ICL V4c implantation.

The miotic agent was used for every eye implanted with the ICL V4 IOL to allow for the flow of aqueous humor through the peripheral iridotomy. Not every eye implanted with the ICL V4c required a miotic agent because of the presence of the central hole, contributing to the significant difference in pupil size observed between the ICL V4 and ICL V4c groups 1 day postoperatively. The vault after ICL V4c implantation was essentially equivalent to the vault after ICL V4 implantation, suggesting that the presence of the central hole did not significantly affect the postoperative vault. With regards to the short term changes after surgery, we found a significant association between the vault changes and the pupil size changes from 1 day to 1 month after the implantation of both ICL V4 and ICL V4c IOLs, concluding that pupil size change is a critical factor in vault change, which is consistent with previous relevant studies [12–16, 18, 24]. We also found a relatively high vault 1 day after surgery in a select number of eyes with small pupils. We inferred that the residual viscoelastic agent played a significant role in the relatively high vault in these eyes 1 day after surgery, as the vault then decreased as the viscoelastic agent was removed by aqueous humor circulation.

There are several factors that may affect the vault after ICL implantation, as reported by Kamiya et al. [18, 24], including pupil movement, age-related increases in crystalline lens thickness and the fixed position of the ICL haptics. Several studies [12–15] have shown that pupil constriction leads to a significant decrease in central vault under photopic conditions, as it causes ICL movement posteriorly towards the crystalline lens. These findings have been supported by Du et al. [16] who demonstrated similar outcomes during pharmacological accommodation with the use of topical pilocarpine. Additionally, the central thickness of the crystalline lens is well known to increase with age [25–27], and a thicker lens may contribute to anterior protrusion of the front surface of the crystalline lens. Garcia-Feijoo et al. [28] concluded that the ICL haptics are typically finally located at either the ciliary sulcus or the ciliary body. Kojima et al. [21] showed that 35.3 % of phakic IOLs were fixated in the ciliary body, while Choi et al. [29] demonstrated that 64.7 % of phakic IOL haptics were fixated in the ciliary sulcus. When the fixation location is changed from the ciliary body to the ciliary sulcus, the vault may decrease.

In our study, pupil movement demonstrated to be a critical factor in vault change. In line with the results of Seo et al. [30], postoperative vault was associated with preoperative ACD and WTW, horizontal and vertical STS in eyes implanted with ICL V4 and ICL V4c. There were no differences of the preoperative factors correlated with postoperative vault between the ICL V4 and ICL V4c groups, suggesting that the presence of the central hole and the varying sizes of ICL V4 and ICL V4c were not a factor correlated with vault. The size of the ICL pIOLs was determined based on the patient’s WTW distance and ACD, therefore, postoperative vault is likely to be associated with ACD and WTW distance. ICL haptics was placed in the ciliary sulcus and the distance of sulcus to sulcus was highly correlated with the distance of white-to-white, so the vault is associated with the horizontal and vertical STS. However, the ocular axis was not an influence factor of the vault in our study although it may be correlated with the ACD and the space of posterior chamber. Previous studies [31] had showed that a statistically significant positive correlation between axis length and ACD in normal and long eyes(<27.5 mm) but not in extremely long eyes (≥27.5 mm), while our study population were moderate to high myopia with long and extremely long axis (25.01 ~ 32.19 mm). Also, the ocular axis was correlated with ACD in neither ICL V4 nor ICL V4c group in our study (*r* = 0.01, *P* = 0.96; *r* = 0.31, *P* = 0.06, respectively).

There were several limitations in our study. Initially, the ICL diameter was chosen according to the WTW diameter, not according to the sulcus-to-sulcus diameter, which is reported to be a more accurate measure for the selection of ICL size [29]. The second limitation of this study was that we did not measure the crystalline lens thickness and the fixed position of the ICL haptics. Another limitation was that our observation period was short and inadequate to determine long-term changes in vault. Further study with a longer follow-up period after surgery is required to ascertain the vault changes of ICL V4 and ICL V4c and the factors affecting vault in the long-term.

## Conclusions

In conclusion, our results demonstrated the dynamic and natural early changes in vault of eyes in the 1-month period immediately following ICL V4 and ICL V4c implantation and the relevant factors affecting vault. We concluded that pupil size change is a critical factor in vault change, and that preoperative ACD, horizontal corneal diameter, horizontal and vertical sulcus-to-sulcus are contributing factors affecting the vault postoperatively. Overall in both groups, there was a trend for the phakic IOL vaults to increase from 1 day to 1 week after surgery in response to an increase in pupil size, with the vault tending to stabilize from 1 month postoperatively. Even when the vault was very low and the ICL was close to the front surface of the crystalline lens, there was never any need to exchange the lens in the early period after ICL implantation, especially for ICL V4c implantation, as the 360 μm central hole allows for the natural flow of aqueous humor and may also reduce the risk of anterior capsular opacification and cataract formation. In these instances, close observation may be sufficient. In order to reduce the risk of complications following ICL implantation, the relevant contributing preoperative factors should be considered when selecting an ICL design and size, especially in eyes with deeper ACD, larger WTW and STS distances that tend towards higher postoperative phakic IOL vault.

## Additional file

Additional file 1: ICL V4, ICL V4c, respectively. Preoperative parameters, vault, respectively. There are 2 sheets named ICL V4 and ICL V4c, each sheet has 2 excels named preoperative parameters and vault, respectively. All the preoperative parameters of ICL V4 and ICL V4c are recorded in the excel “preoperative parameters” and the postoperative vault and the pupil size and their changes are recorded in the excel “vault”. (ZIP 25 kb)

## Abbreviations

ACD: Anterior chamber depth
ANOVA: Analysis of variance
CCT: Central corneal thickness
CDVA: Corrected distance visual acuity
D: Diopters
ECD: Endothelial cell density
ICL: Implantable Collamer Lens
IOP: Intraocular pressure
K: Keratometry
N: Number of eyes
Nd:YAG: Neodymium - yttrium - aluminum - garnet
OCT: Optical coherence tomography
pIOL: Posterior chamber phakic intraocular lens
SD: Standard deviation
STS: Sulcus to sulcus
UBM: Ultrasound biomicroscopy
UDVA: Uncorrected distance visual acuity
WTW: White-to-white
